# Effects of Routine Checkups and Chronic Conditions on Middle-Aged Patients with Diabetes

**DOI:** 10.1155/2020/4043959

**Published:** 2020-02-20

**Authors:** America E. McGuffee, Kailyn Chillag, Amber Johnson, Regan Richardson, Hallie Williams, Jessica Hartos

**Affiliations:** Department of Physician Assistant Studies, University of North Texas Health Science Center, Fort Worth, TX, USA

## Abstract

*Purpose*. Middle-aged males and females with diabetes are more likely to have poor physical (PH) and mental health (MH); however, there is limited research determining the relationship between MH and PH and routine check-up in diabetic middle-aged adults, especially by gender. The purpose of this study was to determine whether PH and MH status differ by routine check-up in middle-aged (age 45–64) adults with diabetes in the general population. *Methods*. This cross-sectional analysis used data from the 2017 BRFSS conducted by the CDC for adults aged 45–64 who reported having diabetes in Florida (*N* = 1183), Kentucky (*N* = 617), Maryland (*N* = 731), New York (*N* = 593), and Ohio (*N* = 754). Multiple logistic regression by state and gender was used to determine the relationship between MH and PH status and routine check-up while controlling for health-related, socioeconomic, and demographic factors. *Results*. Across states, up to one-half reported good PH (32–50%), over one-half reported good MH (46–67%), and most reported having a routine check-up (87–93%). Adjusted analysis indicated that MH and PH were not significantly related to routine check-up, but both were inversely related to having diabetes plus two other health conditions. *Conclusions*. Overall, routine check-up was not related to good PH and MH in this target population; however, a number of health conditions were inversely related to good PH and MH status. In a primary care setting for this target population, there may be a low to moderate prevalence of good PH and MH and a high prevalence of having a routine check-up and having multiple health conditions. It is recommended to automatically screen this target population for PH, MH, other chronic conditions, and physical activity and treat concurrently.

## 1. Introduction

Through utilization of preventative health examinations and routine checkups, the most common chronic physical and mental health conditions can be prevented, delayed, or treated more effectively [6, 11]. Visits of this type are linked with improved physical and mental health outcomes [4, 5, 14] as well as to improvements in health-related quality of life (HRQoL) and control over diseases [10]. However, research indicates that both engagement in preventative health checks and overall perception of one's health differ based on demographic factors, socioeconomic status, health insurance coverage, lifestyle factors, and disease burden [4, 5, 9, 10, 12–14].

Diabetes is a significant component of the disease burden in the US as it affects up to 30 million adults, and of those, up to 7 million may be undiagnosed (not aware of or did not report having DM) [2]. Because this condition is chronic and progressive, it can lead to other physical and mental health complications over time [10–12, 14] . In addition, those with diabetes report lower HRQoL ratings than do individuals without chronic illnesses [10]. Thus, annual or more frequent checkups would be necessary for persons with diabetes as research indicates that preventative care decreases both the prevalence and progression of this condition [6].

However, research for associations between routine checkups and physical or mental health tends to focus on older adults/Medicare users [4–6], with no studies assessing these relations for differences by gender [9, 12–14] or by diabetes status [4, 7, 8]. Therefore, the purpose of this study is to assess whether physical and mental health differ by receiving a routine checkup in middle-aged males and females with diabetes.

## 2. Methods

### 2.1. Design

This study is a cross-sectional analysis that used data from the 2017 Behavioral Risk Factor Surveillance System (BRFSS) conducted by the Centers for Disease Control and Prevention (CDC) [3]. BRFSS is an annual telephone survey system that uses random digit dialing techniques for both landlines and cell phones across all 50 states in the US and the District of Columbia. This survey gathers data about US adult residents' health-related behaviors, chronic health conditions, and use of preventative services. The CDC compiles all BRFSS data and makes deidentified data available for secondary analysis by researchers. This study was given exempt status by the Institutional Review Board of The University of North Texas Health Science Center.

### 2.2. Sample

The samples in the study include males and females with diabetes between the ages of 45 and 64 in Florida (*N* = 1183), Kentucky (*N* = 617), Maryland (*N* = 731), New York (*N* = 593), and Ohio (*N* = 754) that had data for mental health, physical health, and routine checkup. These states were chosen because they had a large diabetic population and higher rates of fair/poor health status when compared to the other states [1].

### 2.3. Data

The original BRFSS variables for mental and physical health were determined by asking participants to self-report the number of “poor health days” in the past 30 days separately for mental health and physical health. Because these responses were highly skewed toward 0 days in each state and because we wanted to predict “good” health, we reversed and dichotomized these values to represent “yes” (30 days of good health in the past 30 days) or “no” (fewer than 30 days of good health in the past 30 days) separately for “good mental health” and “good physical health.” The factor of interest, routine check-up, was measured as yes/no to having a checkup in the past year.

All models controlled for physical activity, weight status, tobacco use, alcohol use, education level, income level, employment status, age, ethnicity/race, and marital status. Health conditions were measured as the number of “yes” responses to whether participants had any of the following: heart attack, coronary heart disease, stroke, asthma, skin cancer, cancer, COPD, arthritis, depression, kidney disease, and diabetes. The resulting numbers were then categorized as “diabetes only,” “diabetes plus one other chronic condition,” and “diabetes plus two other chronic conditions.” Alcohol use was measured in BRFSS as number of drinks per day and we categorized the numbers as “none,” “light” (<1), and “moderate or excessive” (1–4+ females, 1–5+ males). All variables and their categories are shown in Table 1(a).

### 2.4. Analysis

Frequency distributions by state were used to describe the samples. Multiple logistic regression was conducted by state and gender to determine the relationship between mental and physical health status and routine check-up while controlling for health-related, socioeconomic, and demographic factors. The state and gender data were analyzed separately to determine the relationship between the variables across multiple similar samples within our population of interest. As such, similar results in three out of five states were considered reliable evidence for relations. Any observations with missing data for any variable were excluded from the adjusted analysis and all analyses were conducted in STATA Version 15.1 (Copyright 1985–2017 StataCorp LLC).

## 3. Results

### 3.1. Participant Characteristics: Males with Diabetes

Table 1(b) lists participant characteristics for middle-aged males with diabetes. The majority of participants had attended a routine medical checkup within the past year (87–90%) and reported good mental health (56–67%), but less than half reported good physical health (37–50%). Regarding health conditions, most participants had diabetes plus two or more chronic illnesses (71–84%) and were obese (54–63%), yet less than half reported being inactive (33–47%). Regarding health behaviors, one-third to one-half of participants did not use tobacco products (34–55%) or alcohol (47–69%). For demographics, the majority of participants were married (48–60%) and reported their race as white (56–87%). As for socioeconomic status, the majority of respondents had not graduated from college or technical school (67–77%), approximately half were employed (40–59%), and up to two-thirds earned an income of $50,000 or more (40–69%).

### 3.2. Participant Characteristics: Females with Diabetes

Table 1(a) lists participant characteristics for middle-aged females with diabetes. Most participants reported having a routine checkup within the past year (87–93%). About half of the participants reported good mental health (46–55%) and less than half reported good physical health (32–46%). Regarding health conditions, most participants had diabetes plus two or more chronic conditions (80–86%) and were obese (58–68%), and about one-third to one-half reported being inactive (41–55%). Regarding health behaviors, the majority of participants reported no tobacco (48–58%) or alcohol use (55–80%). For demographics, a range of participants reported their race as white (55–82%) and around one half were married (41–52%). As for socioeconomic status, the majority of respondents had not graduated from college or technical school (66–79%) and earned an income of less than $50,000 (50–76%), while one-third to one-half of participants were employed (35–51%).

### 3.3. Mental Health: Males

As shown in Table 2(a), the results of multiple logistic regression analysis for middle-aged males with diabetes indicated that after controlling for all other variables in the model, good mental health was significantly related to having had routine checkups in only 1 out of 5 states, which was not considered a reliable finding across states as was defined in the methods section.

### 3.4. Physical Health: Males

Also shown in Table 2(a), the results of multiple logistic regression analysis for middle-aged males with diabetes indicated that after controlling for all other variables in the model, good physical health was not significantly related to routine checkups across in any state. However, those who reported having diabetes plus two or more other chronic conditions were about 2.5–12.5 times less likely to report good physical health compared to those who reported diabetes only across states.

### 3.5. Mental Health: Females

As shown in Table 2(b), the results of multiple logistic regression analysis for middle-aged females with diabetes indicated that after controlling for all other variables in the model, good mental health was significantly related to having routine checkups in only 1 out of the 5 states, which was not considered a reliable finding across similar samples. However, compared to those with only diabetes, those who reported having diabetes plus two or more other chronic conditions were approximately three to four times less likely to report good mental health compared to those who reported having diabetes only in a majority of the states.

### 3.6. Physical Health: Females

Also shown in Table 2(b), the results of multiple logistic regression analysis for middle-aged females with diabetes indicated that after controlling for all other variables in the model, good physical health was not significantly related to routine checkup across states. However, those who reported having diabetes plus two or more other chronic conditions were about 3–9 times less likely to report good physical health compared to those who reported having diabetes only in the majority of the states.

## 4. Discussion

The purpose of this study was to determine whether physical and mental health status differ by routine checkup in middle-aged males and females with diabetes. For this target population, both good mental and physical health did not significantly differ related to having routine checkups across states. These results differ from prior research which showed that preventative care is related to both improved depressive symptoms and the perception of good overall health [4, 5, 11]. The differences in findings may be due to differing target populations. While our results focus on patients with diabetes, previous research focused on the general population as a whole. Therefore, it is possible that prevention efforts were in place as most of the patients were already under medical care. Thus, to our knowledge, our study is the first to focus only on middle-aged patients with diabetes as related to regular checkups.

However, the results of this study did show that for middle-aged males and females with diabetes, good physical and mental health were inversely related to having diabetes plus two or more other health conditions. This is generally consistent with prior research that showed chronic illnesses to be related to lower overall health in the general population [12]. As prior research focused primarily on the general population, our study focuses solely on middle-aged people with diabetes. For this target population, the comanagement and treatment of multiple chronic conditions may have a significant impact on their mental and physical health, and the assessment of comorbid conditions should be a focus for their care.

### 4.1. Limitations

The use of BRFFS data provided large, multiple samples to evaluate both physical and mental health (instead of “general” health) and separately for males and females (instead of together), which allowed us to determine whether patterns in variable relations were similar for different facets of health and by gender. In addition, BRFSS also provided current information, given that our dependent variables (mental and physical health) were measured within the last 30 days. However, BRFSS did not include variables that provided information about (1) current treatment and management of any mental or physical health condition, (2) current treatment and management of diabetes specifically, or (3) current management and severity of the chronic illnesses in addition to diabetes, all of which could have an impact upon mental and physical health. Future studies should include information about the current management and treatment of any mental and physical health conditions and should consider the influence of specific disease management and medication use in relation to its impact on mental and physical health in diabetics.

## 5. Conclusions

Because this study used population-based data, the results may generalize to males and females aged 45–64 with diabetes. Regarding the optimization of patient's mental and physical health, we recommend automatic mental and physical health screening for adults aged 45–64 with diabetes as well as screening for other chronic health conditions. General practitioners should assess comorbid conditions and treatments, refer to specialists as needed, and educate patients on the importance of proper management of diabetes in combination with any other chronic illnesses for maintaining good mental and physical health.

## Figures and Tables

**Table 1a:** (a) Participant characteristics by state: diabetic males.

Variable	Florida (*N* = 516)		Kentucky (*N* = 236)		Maryland (*N* = 311)		New York (*N* = 300)		Ohio (*N* = 323)	
	*N*	%	*N*	%	*N*	%	*N*	%	*N*	*%*
*Good mental health*	*516*	*100*	*236*	*100*	*311*	*100*	*300*	*100*	*323*	*100*
Yes	333	65	131	56	207	67	199	66	209	65
No	183	35	105	44	104	33	101	34	114	35
*Good physical health*	*516*	*100*	*236*	*100*	*311*	*100*	*300*	*100*	*323*	*100*
Yes	232	45	87	37	154	50	141	47	142	44
No	284	55	149	63	157	50	159	53	181	56
*Routine checkup*	*516*	*100*	*236*	*100*	*311*	*100*	*300*	*100*	*323*	*100*
Yes	451	87	213	90	280	90	268	89	292	90
No	65	13	23	10	31	10	32	11	31	10
*Health conditions*	*475*	*92*	*218*	*92*	*296*	*95*	*286*	*95*	*302*	*93*
0 or 1 other health condition	101	21	34	16	67	23	84	29	55	18
2 other health conditions	374	79	184	84	229	77	202	71	247	82
*Physical activity*	*461*	*89*	*214*	*91*	*270*	*87*	*248*	*83*	*300*	*93*
Inactive	195	42	101	47	90	33	95	38	126	42
Insufficiently active	90	20	48	22	60	22	52	21	62	21
Active/Highly active	176	38	65	30	120	44	101	41	112	37
*Weight status*	*487*	*94*	*230*	*97*	*295*	*95*	*287*	*96*	*315*	*98*
Underweight or normal	53	11	27	12	34	12	32	11	34	11
Overweight	149	31	64	28	83	28	99	34	83	26
Obese	285	59	139	60	178	60	156	54	198	63
*Tobacco use*	*495*	*96*	*229*	*97*	*296*	*95*	*277*	*92*	*315*	*98*
Never	221	45	78	34	149	50	151	55	154	49
Former	182	37	82	36	90	30	85	31	113	36
Current	92	19	69	30	57	19	41	15	48	15
*Alcohol use*	*495*	*96*	*228*	*97*	*298*	*96*	*293*	*98*	*313*	*97*
None	299	60	157	69	146	49	138	47	182	58
Light	57	12	27	12	42	14	46	16	42	13
Moderate or excessive	139	28	44	19	110	37	109	37	89	28
*Age*	*516*	*100*	*236*	*100*	*311*	*100*	*300*	*100*	*323*	*100*
45–54	176	34	76	32	107	34	103	34	83	26
55–64	340	66	160	68	204	66	197	66	240	74
*Ethnicity/Race*	*506*	*98*	*234*	*99*	*304*	*98*	*288*	*96*	*313*	*97*
White	328	65	186	79	191	63	162	56	272	87
Other	178	35	48	21	113	37	126	44	41	13
*Marital status*	*511*	*99*	*235*	*99*	*310*	*100*	*299*	*100*	*321*	*99*
Married	292	57	130	55	187	60	145	48	190	59
Not married	219	43	105	45	123	40	154	52	131	41
*Education level*	*515*	*100*	*233*	*99*	*310*	*100*	*297*	*99*	*321*	*99*
Graduated college	126	25	56	24	209	33	93	31	75	23
Did not graduate college	389	76	177	76	101	67	204	69	246	77
*Income level*	*464*	*90*	*182*	*77*	*272*	*87*	*272*	*91*	*287*	*89*
$50,000 or more	142	31	65	36	164	60	103	38	123	43
Less than $50,000	322	69	117	64	108	40	169	62	164	57
*Employment status*	*512*	*99*	*235*	*99*	*309*	*99*	*291*	*97*	*322*	*99*
Employed	217	42	94	40	181	59	147	51	154	48
Other	295	58	141	60	128	41	144	49	158	52

**Table 1b:** (b) Participant characteristics by state: females with diabetes.

Variable	Florida (*N* = 667)		Kentucky (*N* = 381)		Maryland (*N* = 420)		New York (*N* = 293)		Ohio (*N* = 431)	
	*N*	%	*N*	%	*N*	%	*N*	%	*N*	*%*
*Good mental health*	*667*	*100*	*381*	*100*	*420*	*100*	*293*	*100*	*431*	*100*
Yes	331	50	197	52	232	55	134	46	205	48
No	336	50	184	48	188	45	159	54	226	52
*Good physical health*	*667*	*100*	*381*	*100*	*420*	*100*	*293*	*100*	*431*	*100*
Yes	258	39	123	32	193	46	96	33	162	38
No	409	61	258	68	227	54	197	67	269	62
*Routine checkup*	*667*	*100*	*381*	*100*	*420*	*100*	*293*	*100*	*431*	*100*
Yes	580	87	353	93	372	89	267	91	391	91
No	87	13	28	7	48	11	26	9	40	9
*Health conditions*	*605*	*91*	*355*	*90*	*398*	*95*	*274*	*94*	*395*	*92*
0 or 1 other health condition	115	19	51	14	78	20	52	19	62	16
2 other health conditions	490	81	304	86	320	80	222	81	333	84
*Physical activity*	*610*	*91*	*344*	*90*	*365*	*87*	*250*	*85*	*401*	*93*
Inactive	294	48	189	55	150	41	107	43	198	49
Insufficiently active	116	19	872	21	74	20	5	22	83	21
Active/Highly active	200	33	83	24	141	39	88	35	120	30
*Weight status*	*589*	*88*	*335*	*88*	*378*	*90*	*270*	*92*	*388*	*90*
Underweight or normal	91	15	44	13	4343	11	41	15	31	8
Overweight	147	25	92	27	90	24	73	27	94	24
Obese	351	60	199	59	245	65	156	58	263	68
*Tobacco use*	*643*	*96*	*375*	*98*	*398*	*95*	*275*	*94*	*422*	*98*
Never	325	51	180	48	232	58	143	52	211	50
Former	166	26	108	29	105	26	78	28	111	26
Current	152	24$	87	23	61	15	54	20	100	24
*Alcohol use*	*656*	*98*	*379*	*99*	*410*	*98*	*284*	*97*	*420*	*97*
None	473	72	303	80	227	55	143	60	307	73
Light	68	10	35	9^^^	85	21	78	14	64	15
Moderate or excessive	115	18	41	11	98	24	54	26	49	12
*Age*	*667*	*100*	*381*	*100*	*420*	*100*	*293*	*100*	*431*	*100*
45–54	234	35	140	37	140	33	120	41	134	31
55–64	433	65	241	63	280	67	173	59	297	69
*Ethnicity/Race*	*651*	*98*	*377*	*99*	*416*	*99*	*279*	*95*	*427*	*99*
White	426	65	309	82	230	55	162	58	348	82
Other	225	35	68	18	186	44	117	42	79	19
*Marital status*	*666*	*100*	*379*	*99*	*417*	*99*	*288*	*98*	*429*	*100*
Married	300	45	196	52	200	48	118	41	205	48
Not married	366	55	183	48	217	52	170	59	224	52
*Education level*	*667*	*100*	*380*	*100*	*420*	*100*	*291*	*99*	*429*	*100*
Graduated college	137	21	83	22	142	34	92	32	95	22
Did not graduate college	530	79	297	78	278	66	199	68	334	78
*Income level*	*544*	*82*	*268*	*70*	*357*	*85*	*256*	*86*	*372*	*86*
$50,000 or more	130	24	94	35	179	50	86	34	117	31
Less than $50,000	414	76	174	65	178	50	170	66	255	69
*Employment status*	*662*	*99*	*379*	*99*	*418*	*100*	*287*	*98*	*430*	*100*
Employed	235	36	133	35	213	51	128	45	164	38
Other	427	65	246	65	205	49	159	55	266	62

**Table 2a:** (a) Results of multiple logistic regression analyses across states: males.

Predicting good mental health (yes vs. no)	Florida				Kentucky		Maryland			New York			Ohio		
	*AOR*	95% CI		*AOR*	95% CI		*AOR*	95% CI		*AOR*	95% CI		*AOR*	95% CI	
		Low	High		Low	High		Low	High		Low	High		Low	High
*Routine check-up*															
No	Ref	—	—	Ref	—	—	Ref	—	—	Ref	—	—	ref	—	—
Yes	1.27	0.64	2.50	**4.43**	1.07	18.45	2.06	0.73	5.82	2.02	0.68	5.98	0.95	0.35	2.61
*Health conditions*															
0 or 1 other health condition	Ref	—	—	Ref	—	—	Ref	—	—	Ref	—	—	Ref	—	—
2 other health conditions	**0.24**	0.10	0.54	1.04	0.25	4.25	0.51	0.20	1.28	**0.23**	0.92	0.55	0.78	0 .33	1.84
Predicting good physical health (yes vs. no)	Florida			Kentucky			Maryland			New York			Ohio		
	*AOR*	95% CI		*AOR*	95% CI		*AOR*	95% CI		*AOR*	95% CI		*AOR*	95% CI	
		Low	High		Low	High		Low	High		Low	High		Low	High
*Routine check-up*															
No	Ref	—	—	Ref	—	—	Ref			Ref	—	—	ref	—	—
Yes	0.59	0.29	1.18	2.39	0.53	10.77	1.39	0.50	3.86	2.55	0.84	7.76	1.48	0.54	4.03
*Health conditions*															
0 or 1 other health condition	Ref	—	—	Ref	—	—	Ref	—	—	Ref	—	—	Ref	—	—
2 other health conditions	**0.18**	0.09	0.37	**0.08**	0.01	0.48	**0.41**	0.18	0.96	**0.39**	0.18	0.88	0.95	0.42	2.11

Note.* AOR* = adjusted odds ratio; 95% CI = 95% confidence intervals; Ref = referent group; boldface indicates significance (*AOR*s with 95% CI that do not include 1.00 are significant). All models controlled for physical activity, weight status, tobacco use, alcohol use, age, ethnicity/race, marital status, education level, income level and employment status.

**Table 2b:** (b) Results of multiple logistic regression analyses across states: females.

Predicting good mental health (yes vs. no)	Florida			Kentucky			Maryland			New York			Ohio		
	*AOR*	95% CI		*AOR*	95% CI		*AOR*	95% CI		*AOR*	95% CI		*AOR*	95% CI	
		Low	High		Low	High		Low	High		Low	High		Low	High
*Routine check-up*															
No	ref	—	—	ref	—	—	ref	—	—	ref	—	—	ref	—	—
Yes	1.39	0.71	2.73	1.05	0.31	3.61	0.56	0.22	1.41	**4.58**	1.24	16.91	0.85	0.34	2.10
*Health conditions*															
0 or 1 other health condition	ref	—	—	ref	—	—	ref	—	—	ref	—	—	ref	—	—
2 other health conditions	**0.27**	0.14	0.52	**0.26**	0.09	0.76	**0.35**	0.16	0.77	0.51	0.21	1.29	**0.30**	0.13	0.67
Predicting good physical health (yes vs. no)	Florida			Kentucky			Maryland			New York			Ohio		
	*AOR*	95% CI		*AOR*	95% CI		*AOR*	95% CI		*AOR*	95% CI		*AOR*	95% CI	
			Low		High	Low		High	Low		High			Low	High	Low	High
*Routine check-up*															
No	ref	—	—	ref	—	—	ref	—	—	ref	—	—	ref	—	—
Yes	1.36	0.65	2.87	2.16	0.55	8.49	0.77	0.33	1.80	0.54	0.17	1.71	1.06	0.40	2.77
*Health conditions*															
0 or 1 other health condition	ref	—	—	ref	—	—	ref	—	—	ref	—	—	ref	—	—
2 other health conditions	**0.33**	0.18	0.61	**0.11**	0.04	0.33	0.59	0.30	1.17	**0.14**	0.05	0.39	**0.35**	0.16	0.78

Note.* AOR* = adjusted odds ratio; 95% CI = 95% confidence intervals; Ref = referent group; boldface indicates significance (*AOR*s with 95% CI that do not include 1.00 are significant).

## Data Availability

BRFSS 2017 data is available online from the CDC at https://www.cdc.gov/brfss/annual_data/annual_2017.html.
